# European Pond Turtle (*Emys orbicularis*) Nest Predation: A Study with Artificial Nests

**DOI:** 10.3390/biology12030342

**Published:** 2023-02-21

**Authors:** Jenő J. Purger, Tamás Gergely Molnár, Zsófia Lanszki, József Lanszki

**Affiliations:** 1 BioRes Limited Partnership, Barackvirág utca 27, H-7624 Pécs, Hungary; 2 Department of Ecology, Faculty of Sciences, University of Pécs, Ifjúság útja 6, H-7624 Pécs, Hungary; 3 Department of Applied Fish Biology, Institute of Aquaculture and Environmental Safety, Hungarian University of Agriculture and Life Sciences, Guba Sándor utca 40, H-7400 Kaposvár, Hungary; 4 National Laboratory of Virology, Szentágothai Research Centre, University of Pécs, Ifjúság útja 20, H-7624 Pécs, Hungary; 5 Fish and Conservation Ecology Research Group, Balaton Limnological Research Institute, Klebelsberg Kuno utca 3, H-8237 Tihany, Hungary

**Keywords:** conservation, daily survival rate, egg-laying site, field experiment, Kis-Balaton, marshland, nesting success, red fox, wetland

## Abstract

**Simple Summary:**

Habitat loss and nest predation significantly threaten European pond turtle (*Emys orbicularis*) populations. To reveal predation pressure in a protected area (Kis-Balaton marshland, Hungary) we conducted an artificial nests experiment. We used real nests which had been predated, and near each of them we created a new artificial nest. In each nest hole we put one quail egg, one plasticine egg and turtle egg shells and then covered them and sprayed the surface with water-diluted turtle urine. The majority of the nests were depredated in the first three nights, mostly by red foxes (*Vulpes vulpes*), confirmed by the bite marks preserved on the plasticine eggs, by footprints and excrements found near the nests and by camera recordings. Daily survival rates of quail eggs in artificial nests established in both real and in new nests were similar, suggesting that estimates obtained with artificial nests reflect the degree of predation pressure on real nests. Scattered nests had a lower survival rate than partly scattered and partly linear or only linearly arranged nests. We proved that spraying the nests with diluted turtle urine and marking them with a flag did not affect their survival. The results support turtle nest protection and selective predator control.

**Abstract:**

Nest predation significantly impacts the population decline of the long-living European pond turtle (*Emys orbicularis*). Kis-Balaton is one of the most important habitats of this species in Hungary, and in May 2017 more than 400 damaged nests were counted. To reveal predation pressure, we conducted a study with artificial nests on three sites in this area. On each site, we used 11 depredated real nests, and near each of them, we created new artificial nests; then in every nest we put one quail egg, one plasticine egg and several turtle egg shells. After that, we sprayed the smoothed surface of the covered holes with water-diluted turtle urine, imitating the turtle’s behaviour. Already in the first three nights, 94% of all nests were depredated by the red fox (*Vulpes vulpes*) and in one case by the European badger (*Meles meles*), which was confirmed by the bite marks preserved on the plasticine eggs, by the footprints and excrements found near the nests, as well as by camera recordings. Only 6% of the nests survived during the three weeks of our study. Daily survival rates of quail eggs in artificial nests established in both real (damaged) and in new nests were similar, suggesting that estimates obtained with artificial nests reflect the degree of predation pressure on real nests. On the site where the nests were scattered, their daily survival rate (33%) was significantly lower than on the sites where their arrangement was partly scattered, partly linear (83%), or only linear (76%). On two additional sites, by using simulated turtle nests we showed that spraying the nests with diluted turtle urine and marking them with a flag did not affect their survival, although further methodological testing is needed. The information obtained with artificial nests enables the organization of the protection of the nests of the European pond turtle and selective predator control.

## 1. Introduction

The European pond turtle (*Emys orbicularis* (Linnaeus, 1758)) is a long-living widespread freshwater turtle endemic to the Western Palearctic [1]. European pond turtle populations have shown a considerable decline in recent decades [2], which has been caused by the reduction in suitable wetland habitats. Species associated with wetlands, such as the European pond turtle, have been adversely affected by the regulation of large rivers, the draining of marshes and other wetlands, and the effects of these human interventions have been exacerbated by climate change [3,4,5]. In the last two centuries, the proportion of wetlands in the Carpathian Basin has also decreased drastically [6]. Based on estimates, the European pond turtle population in the marshy part of Kis-Balaton, southwest of Lake Balaton, Hungary’s largest lake, is around a thousand individuals [7]. The dams and embankments created during habitat reconstruction of the Kis-Balaton have become egg-laying places for European pond turtles. However, several potential egg-predator mammal species, such as the red fox (*Vulpes vulpes* (Linnaeus, 1758)) dig their burrows in the dams and search for food on the embankments. Because of that, embankments as linear objects are ecological traps for the turtles that reproduce there [8,9]. Rangers of the Balaton Uplands National Park Directorate and researchers indicated in May 2017 that the number of nests damaged by predators has increased compared to previous years and exceeded 400 on known egg-laying sites in the Kis-Balaton area [10]. In that year, it was impossible to organize the protection of the nests, but there was a chance to determine the level of predation pressure and identify the egg predators. Furthermore, this was a good opportunity to develop an indirect method to estimate the daily survival rate of the European pond turtle.

Most of the nests in the egg-laying sites of the turtles are difficult to find but are detectable efficiently with the help of trained dogs [11]. When examining reproductive success, increased human presence and disturbance also attract predators. Therefore, the use of artificial nests has several advantages, e.g., a study can be carried out at the same time with a larger sample, even at several locations, without negatively affecting the reproduction of the turtles [12,13,14,15]. Furthermore, the experiment can be adapted to the conditions of the egg-laying sites. However, there are also disturbances during the creation of artificial nests (digging, wetting of covered nests, marking with flags) and checking. The use of flags is especially beneficial if the number of samples is large and the arrangement of the nests is random, it shortens the inspection time (disturbance), but the question always arises as to whether this will influence the predation process. The use of artificial nests, despite the several advantages and its simplicity, has not yet been a widespread practice in the study of the predation pressure on the nests of the European pond turtle.

Using this method, our goals were to answer the following questions: (1) when do predation events take place and what is the level of damage; (2) do the daily survival rates of eggs differ in artificial nests, in real, already depredated nests and in new nests created by us; (3) does the arrangement of nests affect their survival; (4) who are the nest predators and (5) whether spraying of the nests with diluted turtle urine and marking them with flags influence predation.

## 2. Materials and Methods

### 2.1. Study Area and Sampling Sites

**Figure 1:**
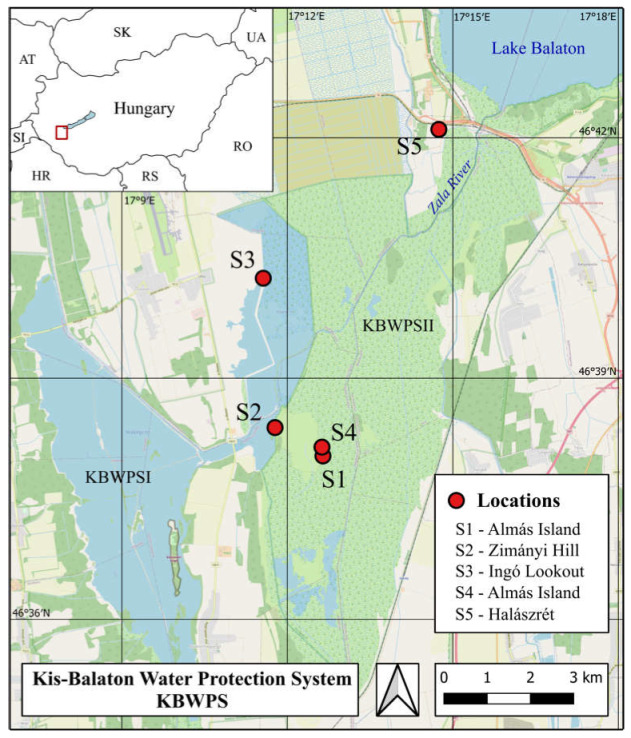
The location of Kis-Balaton and the egg-laying sites of the European pond turtle where the nest predation study was conducted (Site 1–3) and the other two sites where the effects of the treatments on predation were examined (Site 4–5).

Kis-Balaton is situated at the mouth of the Zala River to Lake Balaton, and it is one of the most extensive wetlands in Hungary, with an area of 150 km^2^ [16,17], containing two main parts, KBWPS I and II systems (Figure 1).

This area has become a large wetland and plays an important role in the preservation of species preferring wetland habitats [17]. This strictly protected wetland is part of the Balaton Uplands National Park and the Natura 2000 (SPA and PSCI) network [18], and due to its unique fauna since 1979, has been declared a Ramsar site [7]. The main habitat types in the KBWPS II wetland are the following: open water (10%), swamp vegetation (74%), grasslands (3%), wooded habitats (12%), and built environment (gravel roads, embankments < 1%) [10]. Along the embankments there are known important egg-laying sites of the European pond turtle, from which, at various distances, wetlands with mosaics of shallow water with stands of the lesser pond-sedge (*Carex acutiformis* Ehrh.), reeds (*Phragmites australis* (Cav.) Trin. ex Steud.), and grey willow (*Salix cinerea* L.) can be found [10,19]. In some places, near the embankments, there are sand hills covered with grasslands used by turtles for laying their eggs. Human presence within the study area is very low, including sporadic activities related to habitat conservation and water management [20].

For our study, we have chosen five elevated sites close to the marshy habitats, which have been used for a long time by European pond turtles as their egg-laying sites (Figure 1).

The study area is situated in a region influenced by a continental climate, with some Mediterranean features. In 2017 the average temperature in spring (March–May) and June was 11.4 and 21.3 °C, respectively; the average monthly precipitation in the spring months was only 22 mm, while in June it was 103.2 mm [21].

### 2.2. European Pond Turtle and Its Predators

The breeding season of the European pond turtle usually begins immediately after hibernation, in March or April [22]. Egg-laying lasts from late May to July, rarely to August [23,24]. Some females lay two broods in one season [24,25]. Egg-laying sites are usually within 800 m from water [26]. As soon as a female has found a suitable place to lay her eggs, she starts digging the nest. The female turtle lays the eggs in the hole she dug in the ground, and then covers them with the excavated soil of 5 cm thickness, rubs it smooth with the plastron, while moistening the soil with urine, and finally leaves the nest [26,27]. Egg-laying usually starts after 6.00 p.m. but in cooler weather it will be earlier at 4.00–5.00 p.m., and it usually ends by midnight [26,28,29]. The eggs of the turtles studied in Hungary are 30–41 mm long and 17–23 mm wide, and their shape is an elongated ellipse [30].

The most common potential egg predator on the Kis-Balaton is the red fox. Its relative frequency on the embankments bordering the wetlands in March 2017 was estimated to be one individual every 2 km (J. Lanszki unpublished data). Other less common potential nest predators are the European badger (*Meles meles* (Linnaeus, 1758)), golden jackal (*Canis aureus* Linnaeus, 1758), pine marten (*Martes martes* (Linnaeus, 1758)), Eurasian otter (*Lutra lutra* (Linnaeus, 1758)) and wild boar (*Sus scrofa* (Linnaeus, 1758)). Among the birds, the hooded crow (*Corvus cornix* Linnaeus, 1758) and the magpie (*Pica pica* (Linnaeus, 1758)) are known predators.

### 2.3. Experiment with Artificial Turtle Nests

When designing our nest predation study, we relied on the method used by Hamilton et al. [12] and Marchand et al. [13] in their study on egg predation in the nests of American freshwater turtles. In the study area, we selected three egg-laying sites where at least 22 artificial nests can be created simultaneously without significant disturbance. Before the beginning of the study, we formed artificial eggs similar to turtle eggs by using 10 g of natural plasticine for each of them. We tied a loop-shaped 20 cm long twine to the centre of a 2 cm stick and then placed the stick inside each plasticine egg (Supplementary Materials Figure S1). The prepared 66 plasticine eggs were placed on trays and sprayed with liquid rubber spray (PlastiDip^®^ Blaine, MN, USA) to mask the artificial smell of the plasticine [31]. We dried all the eggs for a day, turned them over, and sprayed the other side. We repeated this procedure two days later. After drying, we placed the plasticine eggs on other trays and stored them in an airy place for two weeks. In our study, we also used real fresh farmed quail eggs (*n* = 66), and prior to the experiment, they were unpacked and stored on paper towels, placed on a tray in the refrigerator and kept in the same ventilated place as the plasticine eggs for three days.

**Figure 2:**
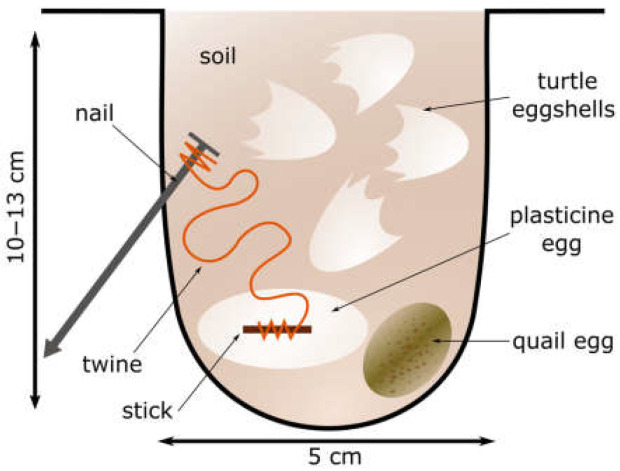
Schematic drawing of artificial turtle nest.

Our experiment with artificial turtle nests started late afternoon on 22 June 2017 and was completed three weeks later on July 13. The locations of the artificial nests were selected following the natural nesting pattern of the turtles. The artificial turtle nests were created at three locations (S1–S3), in each case at a distance of 20–30 m from the wetlands. The nests were 1–10 m apart in all three egg-laying places. In the first egg-laying site (S1), the arrangement of the nests was scattered in patches. In the second egg-laying site (S2), the arrangement of the nests was scattered in patches and linear. Turtles dig their nests in sandy soil in open grassland, on the steep edge of a hill, and in the flat area next to the hill. In the third egg-laying site (S3), the nests were located linearly along the edge of an embankment (Supplementary Materials Figure S2). We used 11 previously depredated real turtle nests per location, and from these, approx. 1–1.5 m away, always in the same direction, but in a similar habitat, we created 11 new nests (S1–S3, 66 nests in total) (Supplementary Materials Figure S2). The artificial nests were made using a hammer and a 5 cm diameter perforated iron pipe. The depth of the drilled holes was 10–13 cm, mimicking the dimensions of an European pond turtle nest [26,27,30]. At the bottom of the hole, a quail egg (as a bait for the predators) was placed [12,32,33], and next to it, one plasticine egg whose primary role was to preserve the predator’s tooth marks [32,33]. To prevent the predator from easily taking the plasticine eggs away from the nest, the twine ending in the loop protruding from the eggs was attached to a 7 cm long, 3.5 mm diameter nail, which was inserted into the inner side of the created holes (Figure 2).

We used rubber gloves to collect empty eggshells from previously depredated nests of European pond turtles, and placed 2–3 pieces above the quail and plasticine eggs in each artificial nest (Figure 2). These dry eggshells have a minimal turtle smell (they were odourless to humans), and we covered them with soil. The turtle nest is small, therefore when a predator scrapes off the eggshells it almost immediately reaches the plasticine and quail eggs, which are real food. The predator comes into contact with a real egg remnants during digging, which maintains its interest in predation and, thus, it is more likely to leave a mark for identification. The use of turtle eggshells as indirect indicators is justified by the fact that eggshells scattered around depredated nests become visually attractive to secondary predators (e.g., birds) [34,35]. In this way, we imitated conditions similar to the predation of real nests. Then, we covered the hole and pressed the sandy soil on top with the palms of our hands. The surface of each nest was then sprayed (approx. 0.20–0.25 dl/nest) with turtle urine diluted with water as explained below [12,13].

A female turtle that had already finished laying eggs was kept for a day to collect its urine in a large tank containing 5 L of water. Before placing the turtle in the tank with water and before releasing it, we kept the animal in a vertical position over the container for a few minutes and observed urination. To minimize human odours, rubber gloves were used during the work, including eggshell collection, digging and shaping the nest, handling and covering the nest material, etc. [12,36].

To make it easier to find the nests in the vegetation during inspections, a 45 cm long thin wooden stick with a 5 × 2 cm white flag was inserted into the ground at a distance of 1 m in the same direction from the artificial nests [37].

The predators were primarily identified based on the tooth and beak marks left on the plasticine eggs [32,33], but footprints left near the nests and fresh predator droppings were also used for identification [38]. We also placed six camera traps at the study sites (3 Dörr BolyGuard 5.0 IR Chesterfield, England, and 3 Minox DTC 450 SLIM, Wetzlar, Germany). In cases when we could not precisely determine the carnivore that robbed the turtle’s nest, we classified it in a medium-sized predator category.

The artificial nests were checked twice a day during the three days following placement, in the morning and in the late afternoon, in order to more precisely determine the time of activity of the predators. On the 8th and 21st days, we performed only afternoon inspections; therefore, estimating the daily survival rates of nests, we used the events recorded during the afternoon inspections. During inspections, we spent as short a time as possible at the sites and collected all materials from the depredated nests and their surroundings. Then, at the last inspection, the artificially placed materials were fully collected.

### 2.4. Testing the Effect of Turtle Urine and Marker Stick

The effect of soil surface wetting and marking of the nests on predation events was studied by simulating turtle nests at two locations (S4, S5). These two known turtle egg-laying sites were 60–90 m from the wetlands and more than 200 m from the other study sites (S1–S3). We simulated 33 nests at both locations so that the 11 simulated nests were placed in three rows at a distance of 1 m from each other (*n* = 66). The nests were imitated by loosening the soil surface to a depth of 3–5 cm in an area with a diameter of 5 cm, and then, similarly to the previous study, we smoothed the surface with our palms. No eggs were placed in such simulated nests. In a random arrangement, three treatments were applied in equal proportions: 1—spraying with diluted turtle urine, 2—spraying with clean water, and 3—placing a marking stick 15 cm from the simulated nests without spraying (Supplementary Materials Figure S3). After the designation of the nests in these two locations (23 June 2017), we checked them for two days in the morning and evening. The simulated nests were considered “predated” if the predator had excavated the soil surface.

### 2.5. Data Analysis

Artificial turtle nests were only considered predated if the quail eggs were damaged or missing from the nest [39]. To estimate the daily survival rates (DSR) of quail eggs, we used Mayfield’s method [40], which is based on the exposure time of nests (the cumulative number of days of artificial nests that survived) and the number of known predation events, and shows the probability that a given nest will remain intact for a day. The DSR values obtained at different locations were compared using the J-test, software developed by K. Halupka [41,42]. The effect of turtle urine and the nest marking flag was evaluated using the chi-square test. All the statistical analyses were performed using SPSS statistical software 25.0 version of IBM SPSS Statistics, Armonk, NY, USA. For all applied statistical tests, *p* < 0.05 was the limit of significance.

## 3. Results

### 3.1. Predation on Previously Depredated Real and New Artificial Nests

In the first three locations, 94% (*n* = 62) of all artificially created turtle nests (*n* = 66) were depredated (the quail eggs were eaten by predators) during the three nights following their placement (Supplementary Materials Figure S4). The remaining 6%, i.e., four nests, were intact even on the 8th and 21st days of the study. Significantly more (chi-square = 7.515, df = 1, *p* = 0.006) quail eggs (*n* = 62) were damaged than plasticine eggs (*n* = 35).

The daily survival rate (DSR = 0.673 ± 0.047 SE, 95% c.l. = 0.579–0.768) of quail eggs (*n* = 33) placed in previously depredated turtle nests did not differ significantly (Z = 1.757, *p* = 0.079) from quail eggs (*n* = 33) placed in new artificial nests (DSR = 0.778 ± 0.036 SE, 95% c.l. = 0.706–0.849). The power test, with *p* = 0.319 (F = 1.008, df = 1) showed an 84% probability of a type II error. Based on the non-significant differences (type I errors), the two nest types were combined in further analyses. As a result, based on the survey conducted at the three egg-laying sites (*n* = 66), we can conclude that the daily survival rate of European pond turtle nests at Kis-Balaton is DSR = 0.728 ± 0.029 SE. This means that a nest has a 73% chance of surviving a day, regardless of the length of the incubation period.

### 3.2. The Predation Depends on Spatial Distribution of Nests

We found that the arrangement of the nests influenced the degree of predation. In the first egg-laying site (S1), the nests were scattered; their daily survival rate of 33% (DSR = 0.333 ± 0.082 SE, 95% c.l. = 0.169–0.497) was significantly lower (Z = −5.548, *p* < 0.001) than that of nests at the second (S2) and (Z = −4.583, *p* ˂ 0.001) third (S3) egg-laying sites (Supplementary Materials Figure S2). At the second egg-laying site (S2), where the nests were both scattered and linear, their daily survival rate of 83% (DSR = 0.829 ± 0.036 SE, 95% c.l. = 0.758–0.901) was similar (Z = 1.162, *p* = 0.245) to the third egg-laying site (S3) where the arrangement of nests was linear and the daily survival rate was 76% (DSR = 0.763 ± 0.045 SE, 95% c.l. = 0.672–0.853). At these two egg-laying sites (S2 and S3), the estimated daily survival rates were higher since the four nests (*n* = 3, *n* = 1) remained intact until the end of the study. The power test, with *p* = 0.029 (F = 3.76, df = 2) showed an 33% probability of a type II error.

### 3.3. Identified Turtle Egg Predators

The tooth imprints preserved in the plasticine eggs (Supplementary Materials Figures S5 and S6), the droppings and other traces (footprints, scraping, and paw nail marks) found near the nests, and the recordings made by the cameras also confirmed that in the majority of cases red foxes robbed the nests (Supplementary Materials Figure S7). We found 18 plasticine eggs with red fox tooth prints, and one with a badger tooth print. When the predator could not be identified based on the tooth imprints (*n* = 14), droppings or footprints around the nest, it was considered a medium-sized carnivorous mammal (Supplementary Materials Figure S5). Only two plasticine eggs were missing from the nests. The morning nest inspections proved that the predation events here always happened at night.

### 3.4. The Effect of Turtle Urine and Marker Stick on Predation Events

We found that neither spraying the nests with diluted urine (chi-square = 0.818, df = 1, *p* = 0.366) nor marking them with flags (chi-square = 3.267, df = 1, *p* = 0.071) had a significant effect on predation events. The egg predators only attempted to rob the simulated nests on the first night at both study sites. The surfaces of 21 nests at site S4 and only one at site S5 were scraped. At both sites, a total of seven (31.8%) nests sprayed with diluted turtle urine (*n* = 22), four (18.2%) nests sprayed with water (*n* = 22), and 11 (50%) nests marked with a flag (*n* = 22) were excavated by predators. Red fox footprints were identified on the surface of the nest in 6 of the 22 predation events.

## 4. Discussion

### 4.1. High-Rate Nest Predation in the First Days after Egg Laying

With our three-week study we showed that 94% of artificial nests made imitating the egg-laying habits of the turtle were depredated in the first three nights after placement. An extremely high predation rate (99.4%) was previously observed at another location in Hungary, where predators discovered the majority of 176 real nests in the first few days following egg-laying [43]. The results of studies on the nesting success of European pond turtles [44] and other turtle species have confirmed that nest predation is most intense in the first week after egg-laying [45]. The reason is that the signals that draw attention to the presence of the nest are the most conspicuous and the most intense in the egg-laying period [46]. Such visual signs include the female turtle laying eggs [47,48], the disturbance of the soil surface [49,50,51,52,53], or the scent of oviposition fluid or urine [15,49,54]. After the start of egg-laying, more female turtles arrive at the egg-laying site, which can attract even more attention from predators [48].

### 4.2. Predation Rates in Previously Depredated Real and New Artificial Nests Was Similar

We showed that the daily survival rate of quail eggs in the artificial nests we created in the same place as depredated real turtle nests were similar to the DSR of quail eggs in the new artificial nests created nearby. Investigating the nest predation of diamond-backed terrapins (*Malaclemys terrapin* (Schoepff, 1793)), Czaja et al. [55] found that the predation rate was similar in the case of both artificial and natural nests. Furthermore, the loss was reduced if the turtles built their nests on rainy days. As a result, the degree of predation pressure on real nests can be well estimated by using artificial nests in a given egg-laying place. Due to the probability of a high type II error, further tests with larger sample sizes should be performed to more precisely reveal the differences between the predation events of real and artificial nests. According to Pärt and Wretenberg [39], artificial nests may only predict the risk for real nests when the nest predator species of the two types of nests are similar. The use of this method has been widespread in ornithological research since the start and length of the study, the number of nests, and their arrangement can be designed according to the conditions of the environment and the nesting habits of the species. In addition, studies with artificial nests are easily repeatable and less time-consuming than tests with real nests [32,56]. Despite these advantages, nest predation studies with artificial nests are not common in turtles [46,55], and rare in the case of the European pond turtle [15]. Our results showed that the survival chance of the turtle nests could be estimated even in a week without disturbing egg-laying turtles. A further reduction in the level of disturbance can be achieved if the study of predation pressure is carried out before the beginning of the egg-laying period, in areas further away from the already known egg-laying places but with similar characteristics.

### 4.3. Spatial Distribution of Nests Affected Predation Rates

Contrary to our expectations, we found a significantly higher daily survival rate of nests in the egg-laying site where their arrangement was scattered and linear or linear than those with the random arrangement. Some studies suggest that female turtles may use olfactory cues to detect the odour of other egg-laying females to select a nest site [57]. Consequently, nest sites tend to have several nests close to each other. Such clumped nests were depredated at a greater rate than scattered nests [13]. We suppose that the differences may not lie in the arrangement of the nests but rather in the activity of the egg-laying turtles and the frequency of occurrence of predators. It is possible that most of the nests which remained intact, were made by turtles at the beginning of the egg-laying period when the predators had not yet started to search for nests. The nesting period is a turning point in turtle activity, it is more limited to the noon and evening hours and does not show temperature dependence [58]. Before egg-laying, their activity is greatest in the morning due to food acquisition and linearly follow the temperature change. During egg-laying, their activity has two peaks, as they dig nests in the evening [58]. In this period, changing from an aquatic to a terrestrial environment [59] can be a significant signal for predators, which presumably start searching for nests based on the change in the turtles’ daily activity. The predators may have been able to notice the turtles or the disturbed ground more easily when the nests were closer to each other, but they could also perceive the smell more strongly.

### 4.4. Red Fox Is Identified as Dominant Predator of Turtle Eggs

Based on the tooth marks preserved on the plasticine, artificial turtle nests were mainly depredated by red fox and, in one case, by European badger. In cases where the predator could not be identified based on the teeth marks, the camera recordings and other marks found at the nests (footprints and excrement) indicated that red foxes also damaged these nests. Quail eggs were damaged to a greater extent than plasticine eggs, which suggested that the predators were the same individuals and probably realized that plasticine eggs were not edible. Predation events occurred during the night when these predatory mammals are more active [60]. Close to our study site, in May and June 2015, the activity of red foxes raising cubs was studied, and 60% of the camera recordings were made at night (É. Fejes, and J. Lanszki, unpublished data). From the large-scale predation, we can conclude that turtle eggs may play an important role in the red fox’s diet, even if this food source is only available for a short time. Female red foxes carry away and cache the larger size sea turtle eggs periodically to provide for their young [61]. From the excrement samples of predatory mammals, egg consumption can only be proven if they accidentally swallow eggshells [62,63], so their consumption is underestimated due to methodological limitations. During the study of the predatory mammals (red fox, mustelids including Eurasian otter) conducted in Kis-Balaton in the period 2014–2020 including egg-laying seasons, turtle eggshells were found in a predator’s excrements in only one case [20]. At fishponds surrounded by forests near our study area, the estimated biomass of consumed turtle eggs accounted for 11% of the badgers’ food [64], but only 0.1% of the food of sympatric red foxes, while turtle egg remains could not be detected in pine marten excrement samples [65]. The red fox and the European badger are considered to be the most significant potential egg predators in the known European turtle egg-laying sites in Hungary [24,30] as well as in the great parts of their distribution range [11,66]. As our results have shown, it is worth using several non-invasive methods to detect egg predators [67]. In addition to plasticine eggs, finding traces preserved on the surface of the soil also helps to identify predators [68]. These traces can be more easily identified in the morning hours, which is why morning nest checks are beneficial. During the day, other, mostly secondary predators can also visit depredated nests and destroy the tracks of the primary predators, or they can deceive us by leaving their tracks behind. In the study area, we observed the hooded crow as a secondary predator at the depredated turtle nests. The rain washes away footprints, so it is not advisable to rely only on this method when identifying predators.

The cameras were used to record not only for the identification of predators but also for the time of predation [15,69,70]. Identifying predators is also important because there may be rare predatory species among them, such as, e.g., Eurasian otter or European wildcat (*Felis silvestris* Schreber, 1775). It is only based on knowledge of egg predators that appropriate loss-reducing measures can be taken for conservation management [49,71,72,73]. European pond turtles are characterized by habitat fidelity and longevity [74,75,76], which is why females try to lay eggs in the same place for several years, despite high predation pressure [77,78]. Turtle nests can be protected with fixed or electric fences, grid panels, repellents [11,79,80], or by removing potential egg predators [71,72].

### 4.5. Cues Moderately Affected Predators to Locate Nests

We found that simulated turtle nests sprayed with water diluted turtle urine were not attractive to predators more than nests sprayed with water alone. This is surprising because the olfactory sense of carnivores, such as the red fox, is sensitive and they rely on it when hunting prey [46,81]. The results of some studies indicate that turtle urine has only a moderate effect on predation events [12,50,69]. Horváth et al. [15] experimentally showed that if only the place of the nest was sprayed with turtle urine diluted with water, it did not affect predation. Still, if the road toward the nest was sprayed with this liquid in larger quantities, it was detected as a signal for predators. Based on these facts, it is conceivable that if the nests are close to each other, then a higher concentration of odorous substances will attract predators; that is, the arrangement of the nests affects predation.

In our study, marking the location of simulated nests with flags did not significantly affect predation events. Several previous studies have also made the same conclusion [37,50,51,82,83], but, e.g., marking of painted turtle (*Chrysemys picta* (Schneider, 1783)) nests with Popsicle^TM^ (Oakland, CA, USA) sticks affected the activity of predators [84]. In our study the flags were not placed close to the nests, and the few centimetre flags on a thin stick were hardly noticeable and could not attract the attention of predators even those with good vision. We proved in our study that red foxes are active during the night [85,86]. Since predation always occurred at night, we presumed that olfactory stimuli played a more significant role in finding nests than visual ones. Spraying the nests with diluted urine and water and marking them with flags attracted egg predators less than disturbing the soil. Scratch marks on the surface of the simulated turtle nests indicated that the red foxes had tried to scrape out the eggs, but as nothing was found, it is likely that they had left the area after a few unsuccessful attempts. In a similar experiment in Slovakia, eggs were also placed in artificial nests, but based on the camera footage, the predators only appeared at the nests on the first night (as in our study), but no predation occurred [15]. Predators seem to be primarily attracted to turtle nests by disturbed soil, possibly via the microbial metabolite geosmin [52]. Experiments with artificial turtle nests showed that the behaviour of predators was not influenced by whether there were eggs in the nest [51,53]. However, the high predation ratio of simulated turtle nests marked with flags suggested that nest marking should be handled with caution and applicability should be verified by further studies in habitats exposed to different human impacts.

## 5. Conclusions

To conserve the European pond turtle populations, it is crucial to know the survival chance of nests in habitats with different predator communities. According to our results, using artificial nests in a week-long study with one check per day, provided enough data about predator pressure. We recommend using real quail eggs placed in the nests to estimate the daily survival rate and plasticine eggs to identify predators. The footprints and excrement found at the site and camera recordings can provide additional information for predator identification. The turtle eggshells that emerge from nests when scraped and scattered by mammal predators attract secondary predators to the nest, such as birds, which have good vision and their predatory activity can only be proven by using cameras. Due to the simplicity of the method, it can be easily applied, and the results obtained form the basis of active nature conservation interventions, e.g., organization of nest protection and predator control.

## Data Availability

Not applicable.

## Supplementary Materials

The following supporting information can be downloaded at: https://www.mdpi.com/article/10.3390/biology12030342/s1, Figure S1: Plasticine eggs sprayed with liquid rubber with twine for fixing them to the ground; Figure S2: Arrangement of the nests in three egg-laying sites. S1—scattered in patches, S2—scattered in patches and linear, S3—located linearly along the edge of an embankment. Gray circles—artificial nests created on the site of a depredated real turtle nest; white circles—newly created artificial nests; Figure S3: Different treatments (⊗ sprayed with water, 🌢 sprayed with diluted turtle urine, ⚐ marked with a flag) of simulated turtle nests. Nests were randomly distributed on the two study sites; Figure S4: Depredated artificial nest with attached plasticine egg and broken quail egg; Figure S5: Distribution of predators identified on the basis of bite marks left on plasticine eggs at three egg-laying sites (S1–S3) in the Kis-Balaton marshland (white—red fox, light grey—badger, dark grey—undetermined medium-sized carnivore, black—plasticine eggs taken away from the nest, n = number of predation events). Figure S6: Red fox tooth imprint preserved on plasticine egg; Figure S7: A red fox is robbing the artificial turtle nest and another fox is seen in the background.

## References

[1] Fritz U. (2003). Die Europäische Sumpfschildkröte (Emys orbicularis).

[2] Fritz U., Chiari Y. (2013). Conservation actions for European pond turtles—A summary of current efforts in distinct European countries. Herpetol. Notes.

[3] Woodward G., Perkins D.M., Brown L.E. (2010). Climate change and freshwater ecosystems: Impacts across multiple levels of organization. Philos. Trans. R. Soc. B.

[4] Markovic D., Carrizo S., Freyhof J., Cid N., Lengyel S., Scholz M., Kasperdius H., Darwall W. (2014). Europe’s freshwater biodiversity under climate change: Distribution shifts and conservation needs. Divers. Distrib..

[5] Albert J.S., Destouni G., Duke-Sylvester S.M., Magurran A.E., Oberdorff T., Reis R.E., Winemiller K.O., Ripple W.J. (2021). Scientists’ warning to humanity on the freshwater biodiversity crisis. Ambio.

[6] Németh G., Lóczy D., Gyenizse P. (2021). Long-term land use and landscape pattern changes in a marshland of Hungary. Sustainability.

[7] Ramsar Sites Information Service Kis-Balaton. https://rsis.ramsar.org/ris/185.

[8] Battin J. (2004). When good animals love bad habitats: Ecological traps and the conservation of animal populations. Conserv. Biol..

[9] Hale R., Swearer S.E. (2016). Ecological traps: Current evidence and future directions. Proc. Royal Soc. B.

[10] Magyari M. (2022). Personal communication.

[11] Schindler M., Frötscher H., Hille A., Bruck M.R., Schmidt M., Kornilev Y.V. (2017). Nest protection during a long-term conservation project as a tool to increase the autochthonous population of *Emys orbicularis* (L., 1758) in Austria. Acta Zool. Bulg..

[12] Hamilton A.M., Freedman A.H., Franz R. (2002). Effects of deer feeders, habitat and sensory cues on predation rates on artificial turtle nests. Am. Midl. Nat..

[13] Marchand M.N., Litvaitis J.A., Maier T.J., DeGraaf R.M. (2002). Use of artificial nests to investigate predation on freshwater turtle nests. Wildl. Soc. Bull..

[14] Marchand M.N., Litvaitis J.A. (2004). Effects of landscape composition, habitat features, and nest distribution on predation rates of simulated turtle nests. Biol. Conserv..

[15] Horváth E., Kaňuch P., Uhrin M. (2021). Predation on nests of the European pond turtle (*Emys orbicularis*): Remarks from failed field experiments. Herpetol. Notes.

[16] Cserny T., Nagy-Bodor E., Gierlowski-Kordesch E.H., Kelts K.R. (2000). Limnogeological investigations on Lake Balaton (Hungary). Lake Basins through Space and Time.

[17] Tátrai I., Kálmán M., Korponai J., Paulovits G., Pomogyi P. (2000). The role of the Kis-Balaton Water Protection System in the control of water quality of Lake Balaton. Ecol. Eng..

[18] NATURA 2000 Standard Data Form—Kis-Balaton (HUBF30003). https://natura2000.eea.europa.eu/Natura2000/SDF.aspx?site=HUBF30003.

[19] Árva D., Tóth M., Mozsár A., Specziár A. (2017). The roles of environment, site position, and seasonality in taxonomic and functional organization of chironomid assemblages in a heterogeneous wetland, Kis-Balaton (Hungary). Hydrobiologia.

[20] Lanszki Z., Horváth G.F., Bende Z., Lanszki J. (2020). Differences in the diet and trophic niche of three sympatric carnivores in a marshland. Mammal Res..

[21] Mandl É. (2022). Personal communication.

[22] Farkas B., Kovács Z. (2008). Noteworthy facts about the European pond turtle. Past, Present, Future of the European Pond Turtle.

[23] Zuffi M.A.L., Odetti F. (1998). Double egg-deposition in the European pond turtle, *Emys orbicularis*, from central Italy. Ital. J. Zool..

[24] Kiss I., Erdélyi G., Szabó B. (2021). Nesting activity and reproductive success of *Emys orbicularis* in Babat Valley (Gödöllő, Hungary). Herpetol. Conserv. Biol..

[25] Zuffi M.A. (2004). Conservation biology of the European pond turtle, *Emys orbicularis*, in Italy: Review of systematics and reproductive ecology patterns (Reptilia, Emydidae). Ital. J. Zool..

[26] Novotný M., Danko S., Havas P. (2004). Activity cycle and reproductive characteristics of the European pond turtle (*Emys orbicularis*) in the Tajba National Nature Reserve, Slovakia. Biologia.

[27] Rogner M., Rogner M. (2009). Nesting. European Pond Turtle Emys Orbicularis.

[28] Meeske M. (1997). Nesting ecology of European pond turtle (*Emys orbicularis*) in South Lithuania. Acta Zool. Litu..

[29] Mitrus S., Zemanek M. (2000). Distribution and biology of *Emys orbicularis* (L) in Poland. Stapfia.

[30] Marián M., Szabó I. (1961). Contribution to the biology of propagation of the tortoise *Emys orbicularis* L. Állat. Közlem..

[31] Purger J.J., Kurucz K., Tóth Á., Batáry P. (2012). Coating plasticine eggs can eliminate the overestimation of predation on artificial ground nests. Bird Study.

[32] Major R.E., Kendal C.E. (1996). The contribution of artificial nest experiments to understanding avian reproductive success: A review of methods and conclusions. Ibis.

[33] Bateman P.W., Fleming P.A., Wolfe A.K. (2017). A different kind of ecological modelling: The use of clay model organisms to explore predator-prey interactions in vertebrates. J. Zool..

[34] Loman J., Göransson G. (1978). Egg shell dumps and crow *Corvus cornix* predation on simulated birds’ nests. Oikos.

[35] Byer N.W., Reid B.N., Seigel R.A., Peery M.Z. (2018). Applying lessons from avian ecology to herpetological research: Techniques for analyzing nest survival due to predation. Herpetol. Conserv. Biol..

[36] Wilhoft D.C., Del Baglivo M.G., Del Baglivo M.D. (1979). Observations on mammalian prediation of snapping turtle nests (Reptilia, Testudines, Chelydridae). J. Herpetol..

[37] Tuberville T.D., Burke V.J. (1994). Do flag markers attract turtle nest predators?. J. Herpetol..

[38] Purger J.J., Mészáros L.A. (2006). Possible effects of nest predation on the breeding success of Ferruginous Ducks *Aythya nyroca*. Bird Conserv. Int..

[39] Pärt T., Wretenberg J. (2002). Do artificial nests reveal relative nest predation risk on real nests?. J. Avian Biol..

[40] Mayfield H.F. (1975). Suggestions for calculating nest success. Wilson Bull..

[41] Johnson D.H. (1979). Estimating nest success: The Mayfield method and an alternative. Auk.

[42] Halupka K. J-Test. http://zeb.uni.wroc.pl/halupka/.

[43] Farkas B., Harmos K., Halpern B. (2014). Mocsári teknős fészek-predáció monitorozása és lehetséges védelmi intézkedések tesztelése a Középső-Ipoly-völgyben. Project: Fenntartható természetvédelem magyarországi Natura 2000 területeken, SH/4/8.

[44] Najbar B., Szuszkiewicz E. (2005). Reproductive ecology of the European pond turtle (*Emys orbicularis* Linnaeus, 1758) (Testudines: Emydidae) in western Poland. Acta Zool. Cracov..

[45] Riley J.L., Litzgus J.D. (2014). Cues used by predators to detect freshwater turtle nests may persist late into incubation. Can. Field-Nat..

[46] Geller G.A., Parker S.L. (2022). What are the primary cues used by mammalian predators to locate freshwater turtle nests? A critical review of the evidence. Front. Ecol. Evol..

[47] Congdon J.D., Breitenbach G.L., van Loben Sels R.C., Tinkle D.W. (1987). Reproduction and nesting ecology of snapping turtles (*Chelydra serpentina*) in southeastern Michigan. Herpetologica.

[48] Eckrich C.E., Owens D.W. (1995). Solitary versus arribada nesting in the Olive Ridley sea Turtles (*Lepidochelys olivacea*): A test of the predator-satiation hypothesis. Herpetologica.

[49] Spencer R.J., Thompson M.B. (2005). Experimental analysis of the impact of foxes on freshwater turtle populations. Conserv. Biol..

[50] Strickland J., Colbert P., Janzen F.J. (2010). Experimental analysis of effects of markers and habitat structure on predation of turtle nests. J. Herpetol..

[51] Bernstein N.P., McCollum A., Black R.W. (2015). How do predators locate nests of ornate box turtles (*Terrapene ornata*)? A field experiment. Herpetol. Conserv. Biol..

[52] Geller G.A. (2015). A test of substrate sweeping as a strategy to reduce raccoon predation of freshwater turtle nests, with insights from supplemental artificial nests. Chelonian Conserv. Biol..

[53] Perazzo G.X., Garcez D.K., Trindade C.R., Pereira K.M., Tozetti A.M. (2018). Is the presence of eggs a relevant cue for predators of freshwater chelonian nests?. Neotrop. Biol. Conserv..

[54] Congdon J.D., Tinkle D.W., Breitenbach G.L., van Loben Sels R.C. (1983). Nesting ecology and hatching success in the turtle *Emydoidea blandingi*. Herpetol..

[55] Czaja R.A., Kanonik A., Burke R.L. (2018). The effect of rainfall on predation of Diamond-backed Terrapin (*Malaclemys terrapin*) nests. J. Herpetol..

[56] Báldi A. (1999). The use of artificial nests for estimating rates of nest survival. Ornis Hung..

[57] Iverson J.B., Klondaris H., Angell C.S., Tori W.P. (2016). Olfaction as a cue for nest-site choice in turtles. Chelonian Conserv. Biol..

[58] Marchand T., Le Gal A.S., Georges J.Y. (2021). Fine scale behaviour and time-budget in the cryptic ectotherm European pond turtle *Emys orbicularis*. PLoS ONE.

[59] Cadi A., Nemoz M., Thienpont S., Joly P. (2004). Home range, movements, and habitat use of the European pond turtle (*Emys orbicularis*) in the Rhône-Alpes region, France. Biologia.

[60] Bennie J.J., Duffy J.P., Inger R., Gaston K.J. (2014). Biogeography of time partitioning in mammals. Proc. Natl. Acad. Sci. USA.

[61] Macdonald D.W., Brown L., Yerli S., Canbolat A.F. (1994). Behavior of red foxes, *Vulpes vulpes*, caching eggs of loggerhead turtles, *Caretta caretta*. J. Mammal..

[62] Reynolds J.C., Aebischer N.J. (1991). Comparison and quantification of carnivore diet by faecal analysis: A critique, with recommendations, based on a study of the fox *Vulpes vulpes*. Mamm. Rev..

[63] Burke R.L., Felice S.M., Sobel S.G. (2009). Raccoon (*Procyon lotor*) predation behavior changes affects turtle (*Malaclemys terrapin*) nest censuses. Chelonian Conserv. Biol..

[64] Lanszki J. (2004). Diet of badgers living in a deciduous forest in Hungary. Mamm. Biol..

[65] Lanszki J., Zalewski A., Horváth G. (2007). Comparison of red fox *Vulpes vulpes* and pine marten *Martes martes* food habits in a deciduous forest in Hungary. Wildlife Biol..

[66] Drobenkov S.M. (2000). Reproductive ecology of the pond turtle (*Emys orbicularis* L.) in the northeastern part of the species range. Russ. J. Ecol..

[67] Bravo C., Pays O., Sarasa M., Bretagnolle V. (2020). Revisiting an old question: Which predators eat eggs of ground-nesting birds in farmland landscapes?. Sci. Total Environ..

[68] Long R.A., MacKay P., Ray J., Zielinski W. (2012). Noninvasive Survey Methods for Carnivores.

[69] Dawson S.J., Adams P., Huston R.M., Fleming P.A. (2014). Environmental factors influence nest excavation by foxes. J. Zool..

[70] Caravaggi A., Banks P.B., Burton A.C., Finlay C.M.V., Haswell P.M., Hayward M.W., Rowcliffe M.J., Wood M.D. (2017). A review of camera trapping for conservation behaviour research. Remote Sens. Ecol. Conserv..

[71] Christiansen J.L., Gallaway B.J. (1984). Raccoon removal, nesting success, and hatchling emergence in Iowa turtles with special reference to *Kinosternon flavescens* (Kinosternidae). Southwest. Nat..

[72] Spencer R.J. (2002). Experimentally testing nest site selection: Fitness trade-offs and predation risk in turtles. Ecology.

[73] Chessman B.C. (2021). Introduced red foxes (*Vulpes vulpes*) driving Australian freshwater turtles to extinction? A critical evaluation of the evidence. Pac. Conserv. Biol..

[74] Escoriza D., Franch M., Ramos S., Sunyer-Sala P., Boix D. (2020). Demographics and survivorship in the European pond turtle (*Emys orbicularis*): A 31-year study. Herpetol. Conserv. Biol..

[75] Bona M., Novotný M., Danko S., Burešová A. (2012). Nest site fidelity in the Slovakian population of the European pond turtle *Emys orbicularis*. Amphibia-Reptilia.

[76] Purger J.J., Molnár T.G. (2022). An unexpected recapture of European pond turtle (*Emys orbicularis* Linnaeus, 1758) in the Barcs Juniper woodland (Hungary). Nat. Somogy..

[77] Mitrus S. (2006). Fidelity to nesting area of the European pond turtle, *Emys orbicularis* Linnaeus, 1758). Belg. J. Zool..

[78] Najbar B., Szuszkiewicz E. (2007). Nest-site fidelity of the European pond turtle (*Emys orbicularis* Linnaeus, 1758) (Testudines: Emydidae) in western Poland. Acta Zool. Cracov..

[79] Vilardell A., Capalleras X., Budó J., Pons P. (2012). Predator identification and effects of habitat management and fencing on depredation rates of simulated nests of an endangered population of Hermann’s tortoises. Eur. J. Wildl. Res..

[80] Buzuleciu S.A., Spencer M.E., Parker S.L. (2015). Predator exclusion cage for turtle nests: A novel design. Chelonian Conserv. Biol..

[81] Bocz R., Batáry P., Purger J.J. (2022). Scent, rather than fur pattern, determines predation of mice: An in-the-wild experiment with plasticine mouse models. J. Zool..

[82] Burke R.L., Schneider C.M., Dolinger M.T. (2005). Cues used by raccoons to find turtle nests: Effects of flags, human scent, and diamond-backed terrapin sign. J. Herpetol..

[83] Edmunds S., Kasparov C.N., Yoon J.B., Kanonik A., Burke R.L. (2018). Twelve years later: Reassessing visual and olfactory cues raccoons use to find diamondback terrapin nests. J. Herpet..

[84] Rollinson N., Brooks R.J. (2007). Marking nests increases the frequency of nest depredation in a northern population of Painted Turtles (*Chrysemys picta*). J. Herpet..

[85] Díaz-Ruiz F., Caro J., Delibes-Mateos M., Arroyo B., Ferreras P. (2016). Drivers of red fox (*Vulpes vulpes*) daily activity: Prey availability, human disturbance or habitat structure?. J. Zool..

[86] Roshier D.A., Carter A. (2021). Space use and interactions of two introduced mesopredators, European red fox and feral cat, in an arid landscape. Ecosphere.

