# Supplementary material for: European Pond Turtle (Emys orbicularis) Nest Predation: A Study with Artificial Nests
Source: Biology (Basel). 2023 Feb 21;12(3):342. doi: 10.3390/biology12030342 (PMC10045932; doi:10.3390/biology12030342)
Supplement: Supplementary file 1 [file biology-12-00342-s001.zip › biology-2194357-supplementary.pdf]

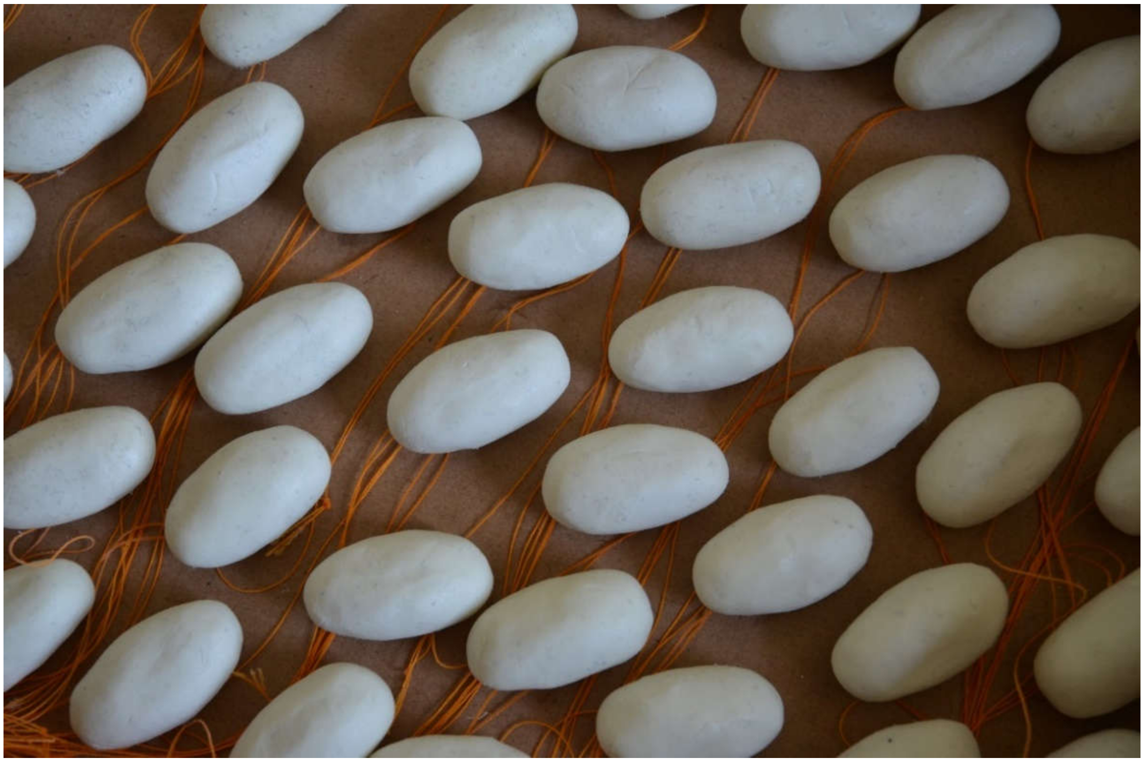

**Figure S1.** Plasticine eggs sprayed with liquid rubber with twine for fixing them to the ground.

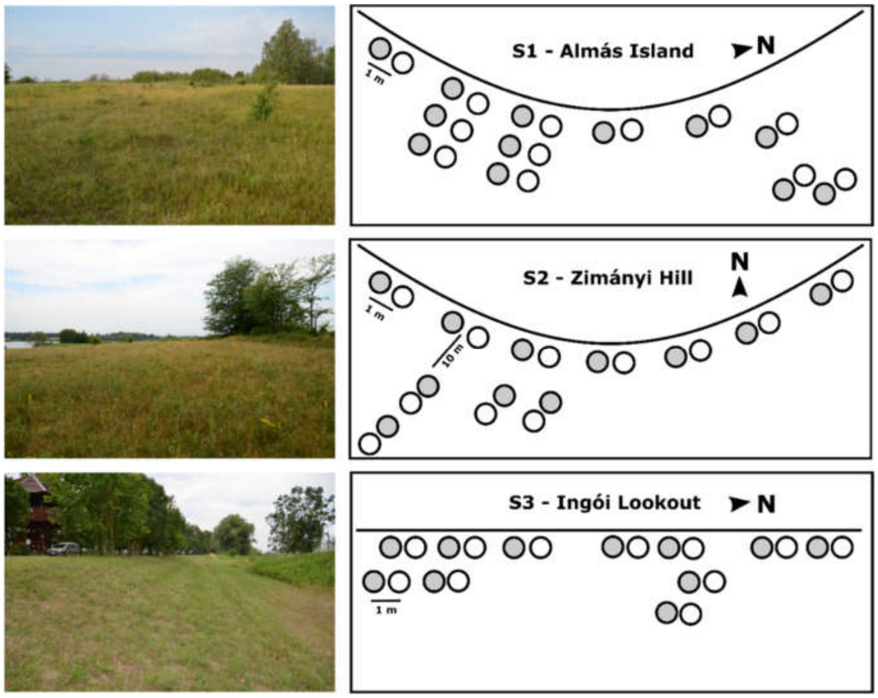


**Figure S2.** Arrangement of the nests in three egg-laying sites. S1 – scattered in patches, S2 – scattered in patches and linear, S3 – located linearly along the edge of an embankment. Gray circles – artificial nests created on the site of a depredated real turtle nest; white circles – newly created artificial nests.

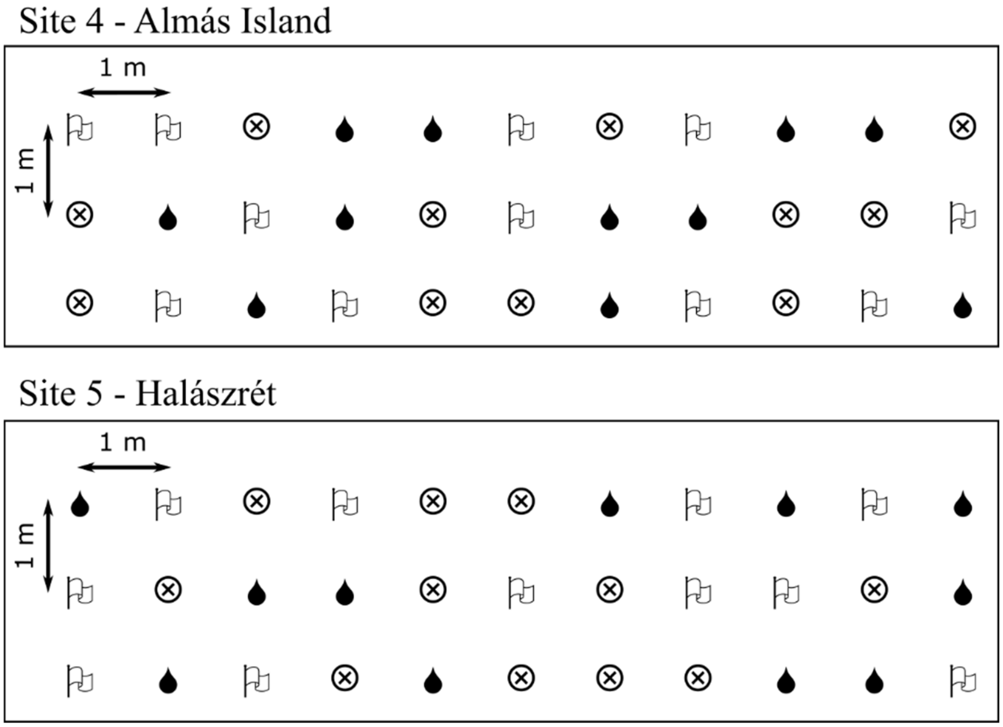


**Figure S3.** Different treatments (⊗ sprayed with water, 💧sprayed with diluted turtle urine, ⚐ marked with a flag) of simulated turtle nests. Nests were randomly distributed on the two study sites.

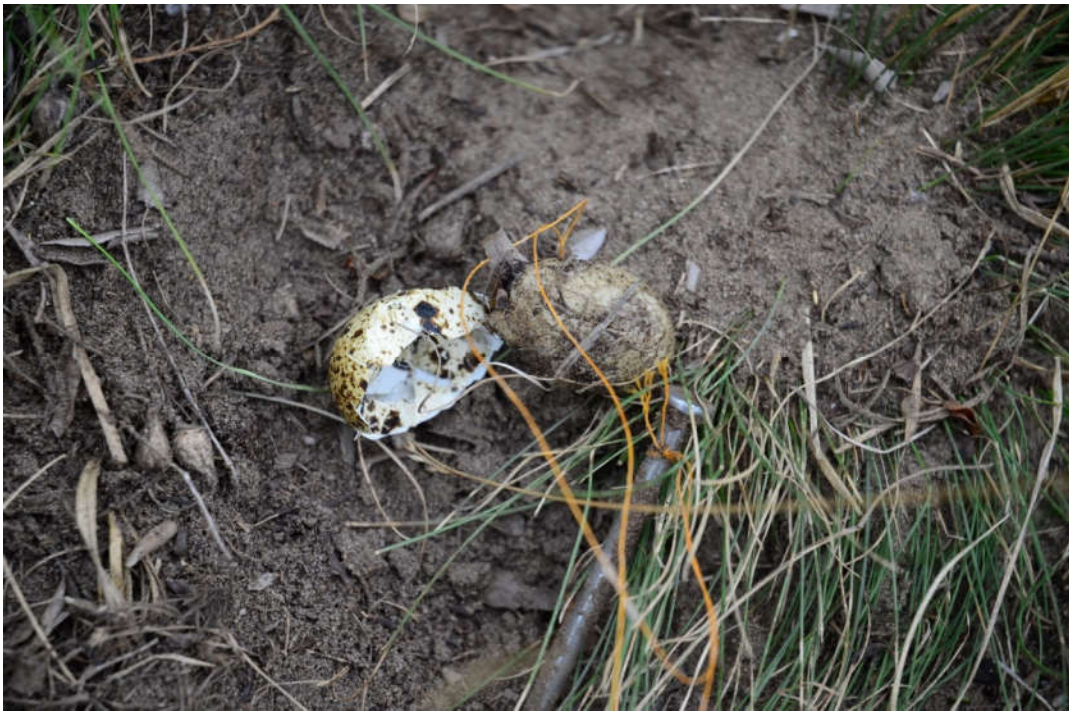

**Figure S4.** Depredated artificial nest with attached plasticine egg and broken quail egg.

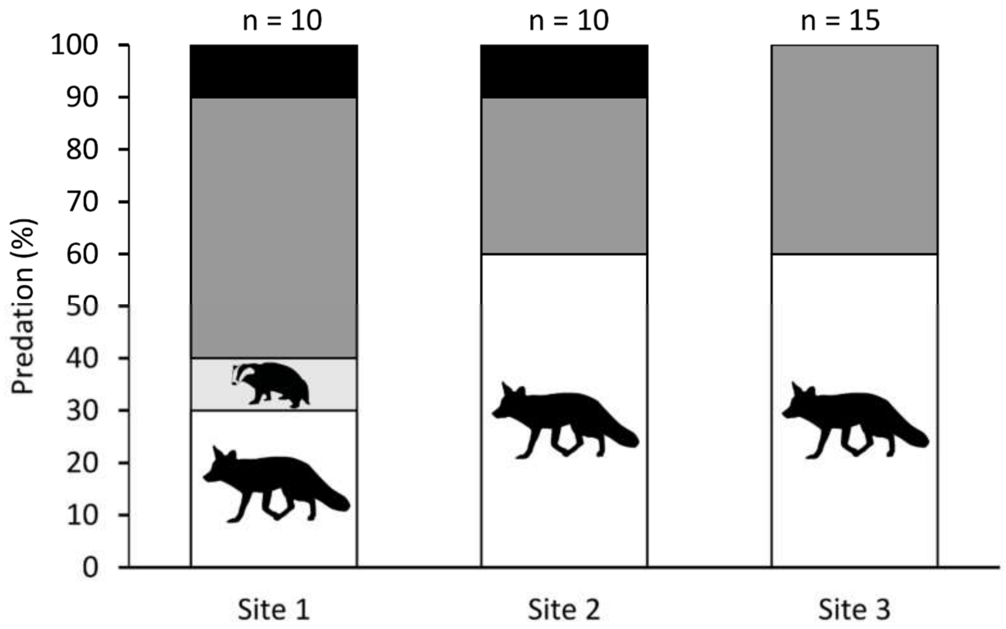


**Figure S5.** Distribution of predators identified on the basis of bite marks left on plasticine eggs at three egg-laying sites (S1, S2, S3) in the Kis-Balaton marshland (white – red fox, light gray – badger, dark gray – undetermined medium-sized carnivore, black – plasticine eggs taken away from the nest, n = number of predation events).

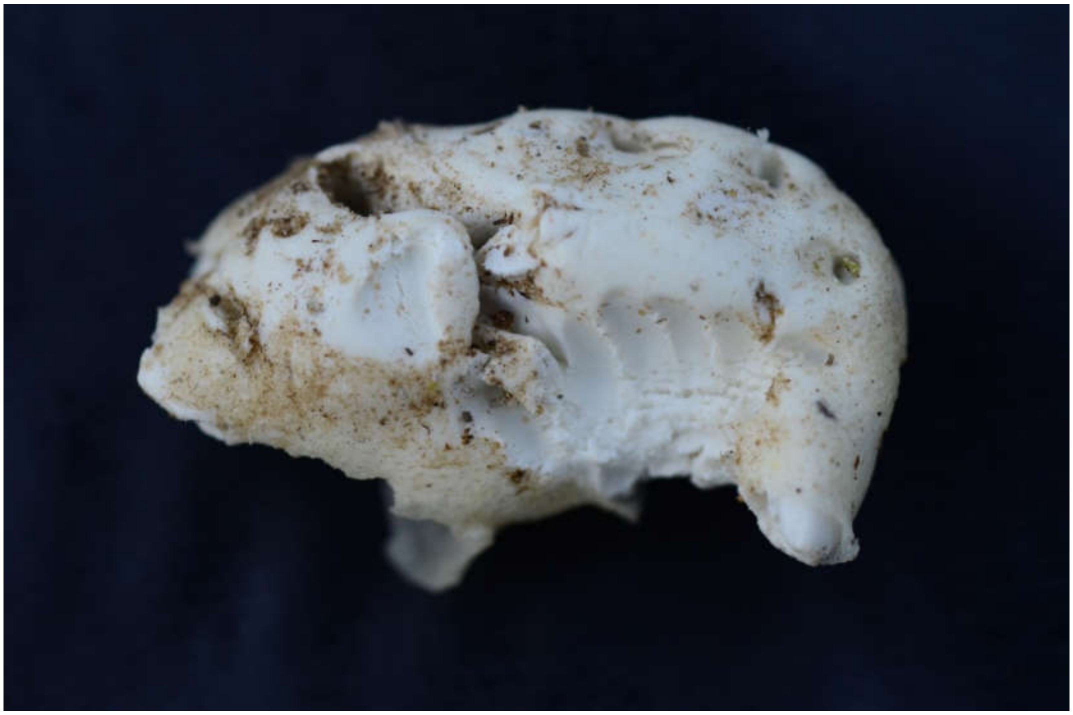

**Figure S6.** Red fox tooth imprint preserved on plasticine egg.

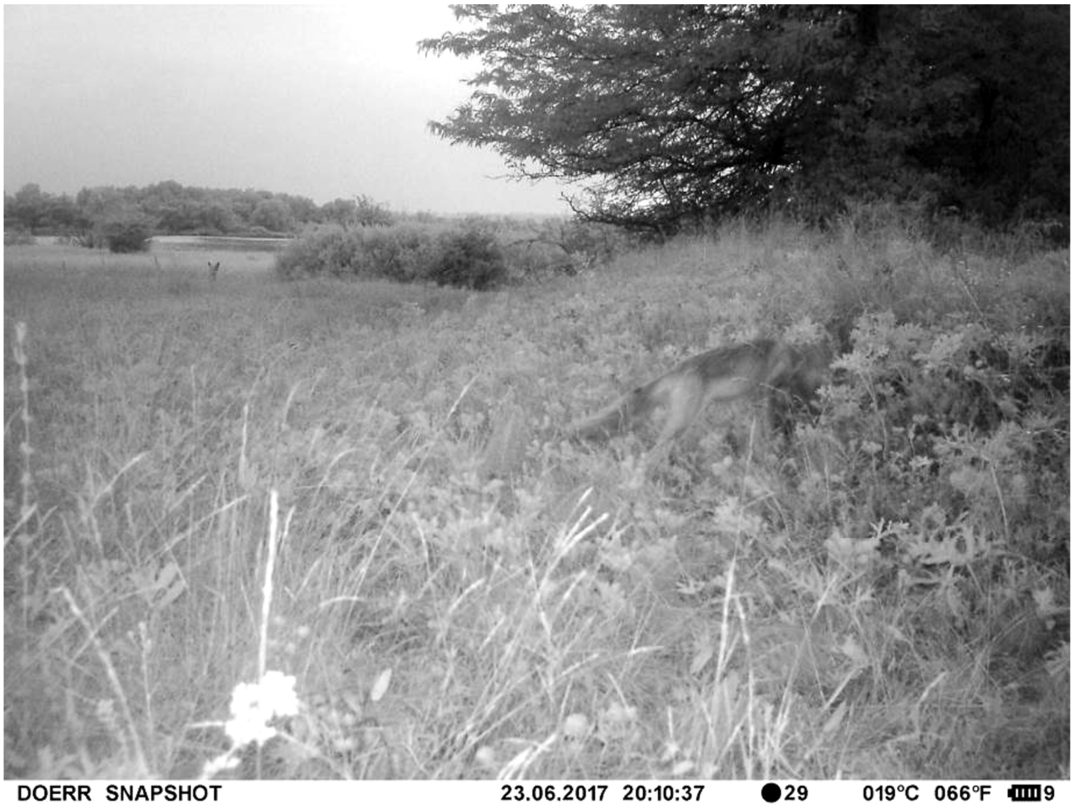


**Figure S7.** A red fox is robbing the artificial turtle nest and another fox is seen in the background.
